# Effect of Methotrexate Injection on Orthodontic Tooth Movement: An Experimental Study on Rats

**DOI:** 10.1155/2021/8451522

**Published:** 2021-11-08

**Authors:** Amin Golshah, Khaled Omidi, Nafiseh Nikkerdar, Hedaiat Moradpoor, Fatemeh Ghorbani

**Affiliations:** ^1^Department of Orthodontic, School of Dentistry, Kermanshah University of Medical Sciences, Kermanshah, Iran; ^**2**^ Student Research Committee Kermanshah University of Medical Sciences, Kermanshah, Iran; ^3^Department of Maxillofacial Radiology, School of Dentistry, Kermanshah University of Medical Sciences, Kermanshah, Iran; ^4^Department of Prosthodontics, School of Dentistry, Kermanshah University of Medical Sciences, Kermanshah, Iran; ^5^Medical Biology Research Center, Health Technology Institute, Kermanshah University of Medical Sciences, Kermanshah, Iran

## Abstract

**Introduction:**

Knowledge about the effects of medications, vitamins, and various supplements on orthodontic tooth movement (OTM) is imperative for orthodontists. This study aimed to assess the effect of methotrexate (MTX) injection on OTM in rats.

**Materials and Methods:**

Twenty-eight male Wistar rats were randomized into four groups (*n* = 7). The first molar and central incisor were connected using a nickel-titanium (NiTi) coil spring with a 50 g load in each rat. The two experimental groups received 0.75 mg/kg and 1.5 mg/kg MTX, respectively, intraperitoneally for 21 days. The negative control group did not receive any injection and did not undergo orthodontic treatment. The positive control group underwent orthodontic treatment and received 0.9% saline (NaCl) injections for 21 days. All rats were sacrificed with chloroform inhalation after 21 days; their maxilla was resected, and the mean number of Howship's lacunae, blood vessels, osteoclasts, and resorption lacunae was counted. The reduction in bone volume (bone volume to total volume ratio (BV/TV)) at the site of the maxillary molar was quantified by microcomputed tomography (micro-CT).

**Results:**

OTM, the number of osteoclasts, and the number of blood vessels significantly increased in rats treated with MTX (*P* < 0.05). However, the increase in the number of Howship's lacunae and resorption lacunae was not significant (*P* > 0.05). Lower BV/TV in the MTX groups was in agreement with the increased number of osteoclasts.

**Conclusion:**

Injection of MTX can significantly increase OTM and decrease root resorption in rats.

## 1. Introduction

Orthodontic tooth movement (OTM) occurs as the result of simultaneous modeling and remodeling of bone and the periodontal tissue. Orthodontic force application also alters the blood vessels in the periodontal tissue and subsequently the blood supply, resulting in local synthesis and release of different molecules [1]. The balanced activity of osteoclasts and osteoblasts plays a fundamental role in this respect [2]. In the process of OTM, osteoclasts are induced to resorb the old bone while osteoblasts are attracted to the areas requiring bone deposition [3]. The proliferation rate of osteoclasts can be used as an important indicator of the level of OTM [4]. The regeneration of periodontal tissue is the basis of OTM. The pressure applied to the alveolar bone induces OTM and tissue regeneration. When the teeth reach their desired position, the alveolar bone and periodontal ligament regain their normal structure [5].

Orthodontic treatment often lasts for 1 to 2 years. Thus, it is imperative for orthodontists to have adequate knowledge about the effects of medications, vitamins, and various supplements on OTM. They should efficiently communicate with patients in this respect, since many patients take different medications for the prevention or treatment of their medical conditions. These medications can reach the periodontal tissue, affected by the orthodontic forces, via the blood circulation and accelerate or decelerate the process of OTM [6]. Nonetheless, information regarding the interactions of medications with OTM is limited, and the risk of such interactions and their adverse effects still exists.

Methotrexate (MTX) was first introduced in the 1940s as a chemotherapeutic agent for the treatment of malignancies. Its favorable anti-inflammatory properties were detected a decade later when physicians started to prescribe it for rheumatoid arthritis patients [7]. MTX has extensive applications for the treatment of malignancies [8]. Its antiproliferative properties are related to its interactions with dihydrofolate reductase, inhibition of DNA synthesis, and prevention of the conversion of folic acid to tetrahydrofolic acid. By doing so, it prevents the participation of folic acid in the synthesis of nucleic acids and proteins. This mechanism of action justifies the use of MTX for conditions with high infiltration rates of inflammatory cells such as T-lymphocytes in the target tissues [7, 8]. On the other hand, clinical investigations have shown that MTX decreases bone growth and bone mineral density and may increase the risk of fractures in older people [9]. In an animal study, osteoporosis occurred in rats under high doses of MTX. Also, the use of MTX has been associated with osteopenia in mice and rats [10–12]. Short-term use of high-dose MTX can be toxic for osteoblasts since it can decrease their volume without changing their number [13]. Moreover, MTX can adversely affect osteoid thickness [11]. Mice and rats are mammals with a metabolism comparable to that of human beings. Thus, they are commonly used to study the cellular and biological mechanisms of OTM [14].

Considering the confirmed effects of MTX on bone formation and lack of studies on its effect on OTM, this study aimed to assess the biological and histological effects of intraperitoneal injection of MTX on OTM in rats.

## 2. Materials and Methods

### 2.1. Animal Model and Study Groups

The study was approved by the ethics committee of Kermanshah University of Medical Sciences (IR.KUMS.REC.1397.724) and was conducted in accordance with the ARRIVE guidelines [15].

This experimental study was conducted on 28 male Wistar rats weighing 200–250 g purchased from Kermanshah University of Medical Sciences. The rats were kept in transparent plastic cages at a constant temperature of 24–25°C, 55% humidity, and 12 h light/12 h dark cycles for 1 week for the purpose of acclimation. The rats were fed soft food to minimize the risk of detachment of the orthodontic appliance after placement [16]. The orthodontic appliance was placed for all rats except for those in the negative control group, as described in previous studies [17, 18]. The rats were randomized into four groups (*n* = 7) by simple randomization:

Experimental group 1: simultaneous with orthodontic treatment, the rats in this group received 0.75 mg/kg MTX injections (Sigma-Aldrich; Saint Louis, MO, USA) intraperitoneally on specific days for 21 days (MTX injections were performed at 1, 5, 9, 13, 17, and 20 days).

Experimental group 2: simultaneous with orthodontic treatment, the rats in this group received 1.5 mg/kg MTX injections (Sigma-Aldrich; Saint Louis, MO, USA) intraperitoneally on specific days for 21 days [19].

The negative control group did not receive any injections and did not undergo orthodontic treatment. The positive control group received 0.9% saline (NaCl) injections intraperitoneally for 21 days simultaneous with orthodontic treatment.

### 2.2. Placement of the Orthodontic Appliance

**Figure 1:**
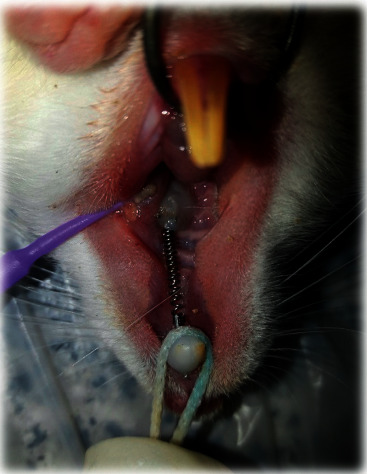
NiTi coil spring placed between the first molar and central incisor of a rat.

The rats were anesthetized by intravenous administration of 10% ketamine hydrochloride (50 mg/kg; Alfasan, Worden, the Netherlands) and 2% xylazine (2 mg/kg; Alfasan, Worden, the Netherlands). After anesthesia induction, the vital signs of the rats were monitored, and they were rolled over every couple of minutes to prevent pulmonary edema. The room temperature was also controlled. Nickel-titanium (NiTi) coil springs (6 mm; G & H Franklin) were placed for induction of OTM. The first molar and central incisor were connected by a stainless steel wire in each rat. The teeth were then etched with 37% phosphoric acid (Vivadent, USA) for 30 s, rinsed for 10 s, and dried with air spray for 15 s. Single Bond (3M ESPE, St. Paul, MN, USA) was applied on the surface of the teeth and light-cured with an LED curing unit (Wood Pecker, Muenster, Germany) with a light intensity of 150 mW/cm^2^ for 10 s. The orthodontic appliance was then fixed by a light-cure composite resin (Transbond XT, 3M ESPE, St. Paul, MN, USA). The load applied by the coil spring was 50 g [20] (Figure 1). To ensure accuracy, the applied load was measured by using a force meter. The lower central incisors were also shortened to prevent possible damage to the orthodontic appliance.

All rats were examined daily to ensure correct position of the coil spring. In case of displacement, the rat would be excluded and replaced [21].

### 2.3. Quantification of OTM

The distance between the distal surface of the first molar and the mesial surface of the second molar was measured before the study onset (day 1) and after 21 days by the same operator blinded to the group allocation of the rats using AB Viewer 14 software. Each measurement was repeated in triplicate, and the mean value was recorded.

**Figure 2:**
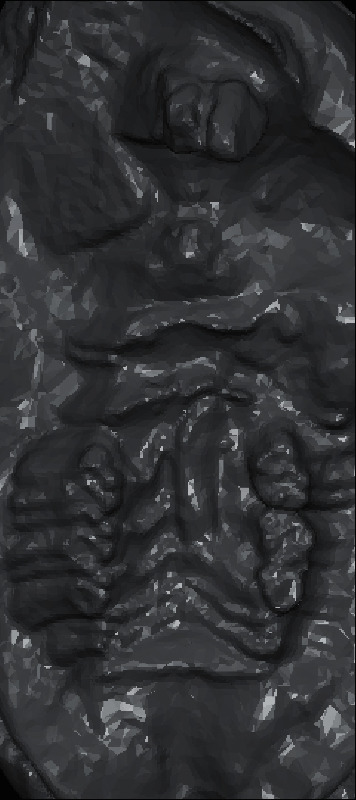
Three-dimensional scan of a sample subjected to MTX and orthodontic force in AB Viewer. The arrow indicates the amount of tooth movement.

Impressions were made of the teeth at 1 and 21 days using polyvinyl siloxane impression material (Express; 3M ESPE, St. Paul, MN, USA); 4 min time was allowed for the material to set, and then, the tray was removed and the impression was poured with a dental stone (Elite Rock Dental Stone; Zhermack, Italy). The cast remained in the impression for 24 h and was then removed. The casts were then scanned by using a 3D scanner (InEos X5; Sirona Dental Systems, Germany) to obtain 3D models in STL format [20, 22] (Figure 2). The STL files were transferred to the AB Viewer software for the measurements.

### 2.4. Histological and Immunohistochemical Analyses

Specimen preparation: after 21 days, all rats were sacrificed in a saturated desiccator by chloroform inhalation. The maxilla was resected for histological analysis. The specimens were fixed in 10% formaldehyde and decalcified in 10% formic acid for 48 h (Sigma-Aldrich, St. Louis, MO, USA). Decalcification was continued in 12.5% ethylene diamine tetra-acetic acid followed by fixation for 10 weeks. The decalcifying solution was agitated 10 times a day and refreshed twice a week until decalcification was completed. Next, all specimens were dehydrated with ethanol and embedded in paraffin blocks. Parasagittal sections with 5 *µ*m thickness were made by using a microtome (Leica, Wetzlar, Germany) [23]. An examiner (A.H.Y) who was blinded to the group allocation of specimens performed the histological analyses.

### 2.5. Histological Analysis

Tissue specimens were stained with hematoxylin and eosin, and the slides were inspected by an experienced pathologist who was blinded to the group allocation of specimens under a light microscope (Eclipse E400, Nikon, Japan) at 100x magnification. The number of Howship's lacunae, blood vessels, osteoclasts, and root resorption lacunae was also counted in an area measuring 0.01 mm^2^. Each specimen was assessed three times, and the mean of the three measurements was recorded.

### 2.6. Micro-CT Assessment

In this study, we used an in vivo X-ray microcomputed tomography (micro-CT) scanner. LOTUS-inVivo has a cone-beam microfocus X-ray source and a flat panel detector. In order to obtain the best possible image quality, the X-ray tube voltage and current were set to 60 kV and 130 µA, respectively, and the frame exposure time was set to 1 second at 2.7 magnification. The total scan duration was 28 minutes. Slice thicknesses of the reconstructed images were set to 50 micrometers. All the protocol settings were controlled by LOTUS-inVivo-ACQ software. The acquired 3D data were reconstructed using LOTUS-inVivo-REC by a standard Feldkamp, Davis, and Kress (FDK) algorithm. Also, LOTUS-inVivo-3D was used for rendering of reconstructed images, and by adding the bone analysis plugin (BAP) inside the software, we reported bone volume (BV) and total volume (TV) parameters [24].

### 2.7. Statistical Analysis

Data were analyzed using SPSS version 21 (SPSS Inc., IL, USA) via one-way ANOVA, Tukey's test, and the *t*-test.

## 3. Results

The minimum intraclass correlation coefficient for all variables was 0.964, which indicated excellent intraobserver agreement.

### 3.1. Effect of MTX on OTM

**Figure 3:**
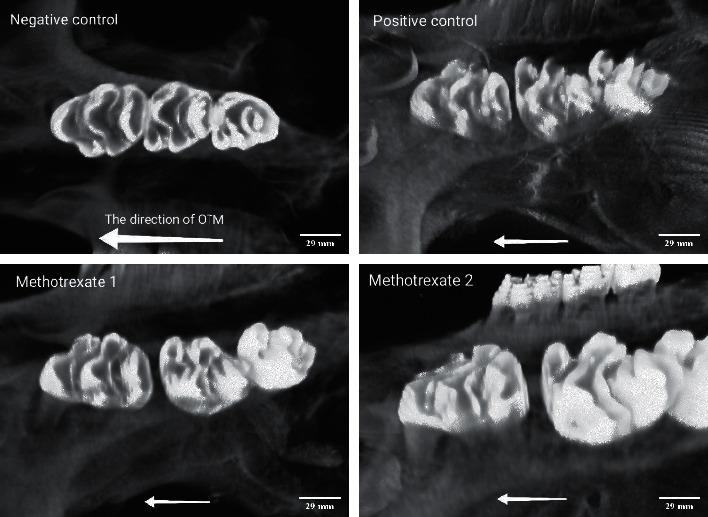
Micro-CT image analysis of orthodontic tooth movement (OTM) after 21 days of force application: the negative control group (no OTM and no injection), positive control group (OTM and saline injection), and experimental groups injected with 0.75 and 1.5 mg/kg methotrexate. Scale bar: 29 mm. Magnification: 2.7.

Figure 3 presents the descriptive data regarding OTM in the four groups. After 21 days of orthodontic force application, OTM in the second 1.5 mg/kg MTX group was significantly higher than that in the other three groups, indicating that MTX injection in 1.5 mg/kg dosage enhanced OTM in rats. No significant difference was noted in this regard between the 0.75 mg/kg MTX group and the positive control group (*P*=0.164). The comparison of other groups revealed no significant difference either (*P* > 0.05, Table 1).

Figure 3 shows micro-CT images of OTM in the four groups.

### 3.2. Histopathological Evaluation

**Figure 4:**
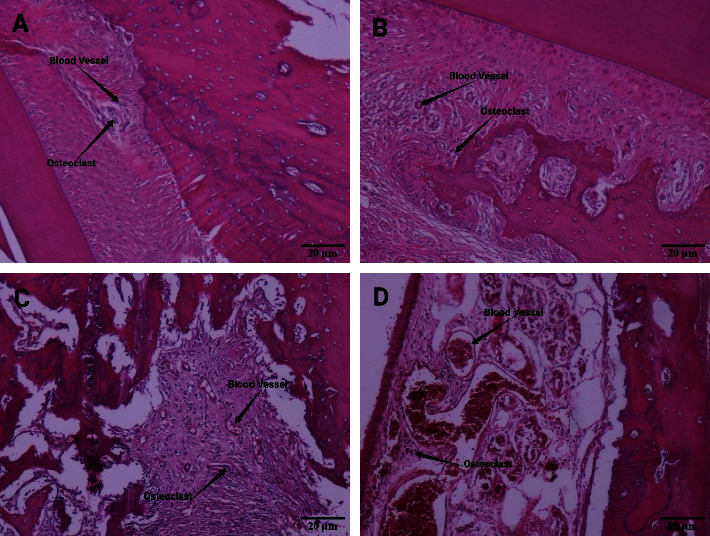
The result of HE staining. Blood vessels and osteoclasts in (a) negative control, (b) positive control, (c) methotrexate 0.75 mg/kg, (d), and methotrexate 1.5 mg/kg. Scale bar 20 *µ*m.

Light microscopic findings of the periodontal tissue showed that, in the negative control group, the number of blood vessels and osteoclasts was histologically normal. In the positive control group, which was affected by orthodontic force, the number of blood vessels and osteoclasts increased on average, but this increase was more pronounced in the two groups of methotrexate, which were simultaneously affected by orthodontic force and different doses of methotrexate (Figure 4).

### 3.3. Histological Analysis

Table 2 shows the mean and standard deviation of histological variables in the groups.

### 3.4. Howship's Lacunae

At 21 days, a significant difference existed among the four groups in the number of Howship's lacunae (*P* < 0.05). The number of Howship's lacunae was maximum in the 1.5 mg/kg MTX group and minimum in the negative control group. The negative control group had a significantly lower number of Howship's lacunae than the other three groups (*P* < 0.05). The positive control group had no significant difference from the MTX groups in this regard (*P* > 0.05). The two MTX groups were not significantly different in this respect either (*P* > 0.05).

### 3.5. Blood Vessels

At 21 days, a significant difference existed among the four groups in the number of blood vessels (*P* < 0.05). The number of blood vessels was maximum in the 1.5 mg/kg MTX group and minimum in the negative control group. The negative control group had a significantly lower number of blood vessels than the other three groups (*P* < 0.05). With an increase in the MTX dosage, the mean number of blood vessels significantly increased such that the difference in this respect was significant between the two MTX groups (*P*=0.03). On the other hand, the number of blood vessels was significantly higher in the positive control group than in the negative control group (*P*=0.03).

### 3.6. Osteoclasts

At 21 days, a significant difference existed among the four groups in the number of osteoclasts (*P* < 0.05). The number of osteoclasts was maximum in the 1.5 mg/kg MTX group and minimum in the negative control group. The negative control group had a significantly lower number of osteoclasts than the other three groups (*P* < 0.05). With an increase in the MTX dosage, the mean number of osteoclasts significantly increased such that the difference in this respect was significant between the two MTX groups (*P*=0.02). The positive control group had no significant difference with the 0.75 mg/kg MTX group in this respect (*P*=0.241). But, the difference was significant between the 0.75 and 1.5 mg/kg MTX groups (*P*=0.026).

### 3.7. Root Resorption Lacunae

At 21 days, a significant difference existed among the four groups in the number of root resorption lacunae (*P* < 0.05). The mean number of root resorption lacunae was maximum in the 1.5 mg/kg MTX group and minimum in the negative control group. No root resorption lacunae were seen in the negative control group. The difference in this regard was significant between the negative control and the other three groups (*P* < 0.05). The two MTX groups (*P*=0.878), the positive control and 0.75 mg/kg MTX (*P*=0.461) and the positive control and 1.5 mg/kg MTX (*P*=0.148) groups, were not significantly different in this respect.

## 4. Effect of MTX on BV/TV

At 21 days, a significant difference existed among the four groups in BV/TV (*P* < 0.05). The BV/TV ratio was maximum in the negative control and minimum in the 1.5 mg/kg MTX group. The negative control group had significantly higher BV/TV ratio than the other three groups (*P* < 0.05). The difference in this regard was not significant between the positive control and 0.75 mg/kg MTX (*P*=0.438) or the two MTX groups (*P*=0.104). But, the difference was significant between the positive control and 1.5 mg/kg MTX groups (*P*=0.003, Table 3).

## 5. Discussion

The application of orthodontic forces to the teeth initiates a cascade of biological events to induce OTM. Several factors can affect this process [25]. In recent years, many studies have assessed the effect of different supplements, vitamins, and medications on OTM [1, 26, 27]. Some materials such as vitamin C and vitamin E have no local or systemic effect on the speed of OTM. Nonetheless, osteoblastic activity was higher at the tension side in all groups that received vitamins. The administration of vitamin C and vitamin E during orthodontic treatment has confirmed positive effects on bone regeneration and can be used to accelerate the process of OTM [28]. Another study showed that injection of asperosaponin VI may enhance OTM by increasing the osteoclastic activity and inducing bone resorption at the pressure side. Moreover, asperosaponin VI has a positive effect on bone formation at the tension side [29]. This study assessed the effect of MTX on OTM in rats. Rats are preferred to study bone regeneration in response to mechanical forces and have been used in the majority of previous studies on OTM [30–32]. This study was the first to show that MTX injection can significantly enhance OTM in rats. MTX is a synthetic antifolate used for the treatment of many conditions such as rheumatoid arthritis, acute leukemia, psoriasis, or even ectopic pregnancy [33]. Animal studies have demonstrated that high-dose MTX can impair the function of the epiphyseal growth plate, degrade osteoprogenitor cells, suppress bone formation, and increase bone resorption. Generally, it can lead to bone loss [33]. Two similar studies showed that low-dose MTX had no significant effect on new periosteal bone formation. Nonetheless, high doses of MTX significantly inhibited bone formation [34, 35]. The current results revealed that orthodontic force can activate osteoclasts and increase angiogenesis. On the other hand, intraperitoneal injection of 0.75 and 1.5 mg/kg MTX simultaneous with the application of orthodontic force further increased the number of osteoclasts and blood vessels and resultantly increased OTM. Moreover, increasing the MTX dosage enhanced OTM. Histological analysis revealed that although MTX injection increased the number of osteoclasts and blood vessels, it did not significantly increase the number of Howship's lacunae or root resorption lacunae. The increase in the number of osteoclasts as the result of injection of MTX was in agreement with previous findings [12, 36, 37]. Micro-CT assessment revealed that the lower BV/TV ratio in MTX groups was in agreement with the increase in the number of osteoclasts. This reduction in the BV/TV ratio can be due to the presence of stainless steel ligature wire around the first molar tooth. The reduction in BV/TV ratio as a result of MTX injection was in accordance with previous findings [38]. Nonetheless, some complex factors may probably affect the measurement of OTM such as deformity of the appliance during mastication, mesial drift of the second molar, and cranial growth during the experiment [21]. It should be noted that all measurements were made by the same operator in this study to prevent interobserver errors. All these factors may vary among different animals, which were not addressed in this study. Nonetheless, these effects should be taken into account in long-term studies. It should be noted that all measurements were made by the same operator with excellent intraobserver agreement in this study, which was a major strength.

## 6. Conclusions

The current results showed that MTX can enhance OTM and decrease root resorption. Further studies are required to analyze the mechanisms involved in this process. Generalization of the results of animal studies should be carried out with caution, and the results should be confirmed in clinical trials. Given that the results are confirmed, orthodontists should pay attention to the effects of MTX on bone in orthodontic treatment planning for their patients.

## Figures and Tables

**Table 1:** Mean and standard deviation of orthodontic tooth movement (mm) in the four groups.

Groups	Distance of orthodontic tooth movement (OTM) at the beginning of the study (mm)		Distance of orthodontic tooth movement (OTM) at the end of 21 days (mm)		Differences between the beginning of the study and the end of the study (mm)
	Mean	SD	Mean	SD	Mean
Negative control	0.20	0.02	0.22	0.02	0.02^a^
Positive control	0.25	0.01	0.44	0.03	0.19^b^
Methotrexate 0.75 mg/kg	0.20	0.01	0.51	0.033	0.31^b^
Methotrexate 1.5 mg/kg	0.22	0.03	0.78	0.099	0.56^d^

^†^Welch one-way ANOVA test followed by Tukey's test was used. Means with same superscript letters are not significantly different (*P* > 0.05). SD: standard deviation.

**Table 2:** Mean and standard deviation of each histological variable in the four groups (mm^2^).

Group	Number of Howship's lacunae		Number of blood vessels		Number of osteoclasts		Number of root resorption lacunae	
	Mean	SD	Mean	SD	Mean	SD	Mean	SD
Negative control	0.16	0.40	1.66	0.81	0.83	0.40	0.00	0.00
Positive control	2.00	0.89	3.50	1.04	2.16	0.75	0.83	0.40
Methotrexate 0.75 mg/kg	2.83	0.75	5.50	1.04	3.00	0.89	1.16	0.40
Methotrexate 1.5 mg/kg	3.16	1.47	7.33	1.21	4.33	0.81	1.33	0.51
*P* value‡	<0.001		<0.001		<0.001		<0.001	

‡One-way ANOVA test followed by Tukey's test was used. Means with same superscript letters are not significantly different (*P* > 0.05).

**Table 3:** Mean BV/TV in the experimental and control groups.

Groups	Bone volume to total volume ratio (BV/TV %)	
	Mean	SD
Negative control	74.2	3.6
Positive control	67.7	4.8
Methotrexate 0.75 mg/kg	64.7	2.6
Methotrexate 1.5 mg/kg	60.0	3.1
*P* value‡	<0.001	

‡One-way ANOVA followed by Tukey's test. Means with the same superscript letters are not significantly different (P  >  0.05). SD: standard deviation.

## Data Availability

No data were used to support this study.
